# Unusual Presentation of Rare Case of Papillary Adenofibroma of Cervix in a Young Woman

**DOI:** 10.1155/2012/914642

**Published:** 2012-02-02

**Authors:** H. Mahesha Navada, B. Poornima Ramachandra Bhat, Gayatri Ramani, R. Rohan Chandra Gatty, C. S. Jayaprakash

**Affiliations:** ^1^Department of Obstetrics and Gynecology, Father Muller Medical College, Mangalore-2, Karnataka 575 002, India; ^2^Department of Oncosurgery, Father Muller Medical College, Mangalore-2, Karnataka 575 002, India; ^3^Department of Pathology, Father Muller Medical College, Mangalore-2, Karnataka 575 002, India

## Abstract

Adenofibroma is an extremely rare benign biphasic neoplasm that is classified into the mixed epithelial and mesenchymal tumor group. These tumors tend to occur in postmenopausal and elderly women. We report the case of a large polypoidal mass per vagina occupying the whole pelvis in a young woman. Preoperative biopsy showed benign epithelial and mullerian mesenchymal components suggestive of mullerian adenofibroma. Total hysterectomy with bilateral salpingectomy was done. The diagnosis of papillary adenofibroma of cervix was made. The total surgery assured complete excision and permitted adequate sampling to exclude malignancy.

## 1. Introduction

Uterine adenofibroma was first described by Ober in 1959 as a form of mixed mesodermal tumor [1]. Both the stromal and epithelial components of this tumor is benign [2]. Cervical adenofibroma was first reported by Abell in 1971 [3]. It is a rare neoplasm that accounts for only 10% of uterine adenofibromas, with most tumors arising in the endometrium [4]. Adenofibromas can be seen in women of any age but they occur most frequently in peri- or postmenopausal women [5]. In this paper, a young woman with large adenofibroma of the cervix occupying the whole pelvis, who presented with acute obstructive symptoms, is discussed.

## 2. Case Report

**Figure 1:**
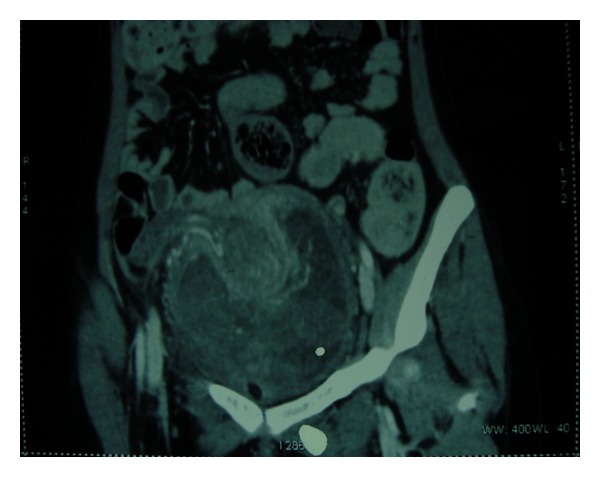
Coronal section computed tomography showing heterogeneously enhancing mass lesion engulfing the uterus, broad ligament and accompanying vessels.

**Figure 2:**
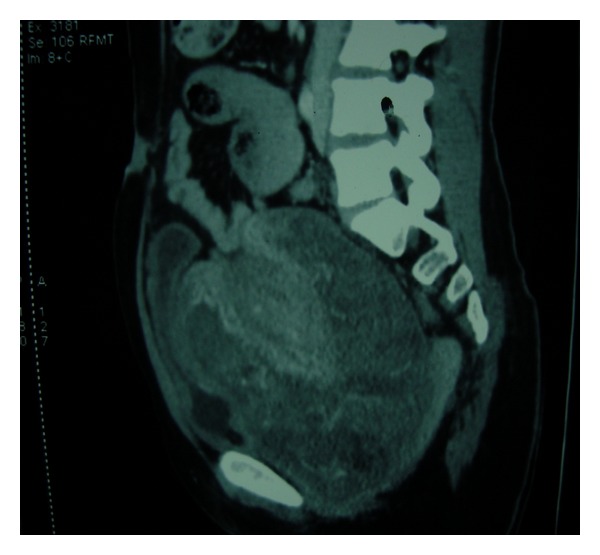
Sagital section computed tomography showing the lesion compressing the bladder anteriorly and the rectum posteriorly maintaining the intervening fat planes.

**Figure 3:**
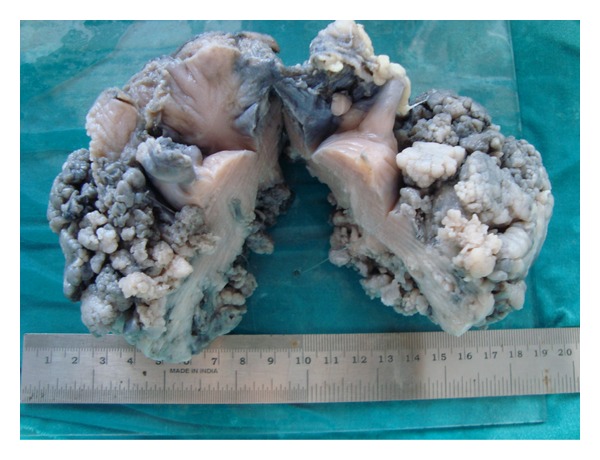
The cut section of the uterus showing large polypoidal mass arising from the cervix, which engulfs the anterior and posterior aspect of the uterus sparing the fundus and tubes.

**Figure 4:**
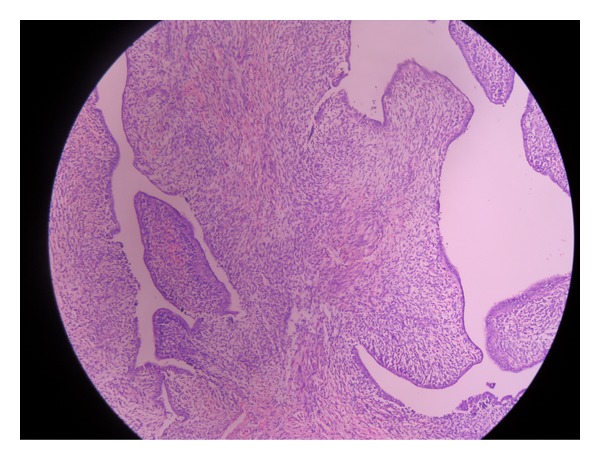
Photomicrograph (H&E stain: original magnification ×10) showing the papillary growth lined by cuboidal epithelium projects from the surface and into the cystic spaces. The subepithelium has scattered glands with surrounding stroma composed of fibroblasts.

A 21-year-old unmarried lady with history of brownish discharge per vagina and lower abdominal pain for the past 2 months presented with acute retention of urine. There was no history of mass per abdomen or mass per vagina. Her last menstrual period was 2 months back. The previous menstrual cycles were regular. There was mild pallor on examination. The abdomen was soft and no mass palpable. Ultrasonography showed large soft tissue lesion embracing the uterus all around. Bilateral ovaries were normal. Computed tomography scan showed a large well-defined heterogeneously enhancing mass lesion measuring 10.4 × 11 × 14 cms. This appeared to arise from the vagina extending superiorly up to the level of the pelvic inlet (Figure 1). The lesion was seen engulfing the uterus and broad ligament and accompanying blood vessels superiorly. It was also seen compressing the bladder anteriorly and the rectum posteriorly (Figure 2). Vaginal examination under anesthesia showed large polypoidal mass distending the vagina and occupying the whole pelvis. The vaginal walls appeared free from the mass. The exact origin of the mass could not be made out as it was not possible to get above the mass. Histopathology of the biopsy taken from the vaginal mass showed benign epithelial and mesenchymal components suggestive of adenofibroma. The tumor markers like alphaetoprotein, CA 125, and beta HCG were within the normal range. The exploratory laparotomy was planned after ureteric stenting. Preoperative ureteric stenting could be achieved only on one side. On the other side ureteric orifice was not accessible due to anatomical distortion by the pelvic mass. Intraoperatively, a large pelvic solid tumor mass was seen burying the uterus. The bilateral tubes and ovaries were seen projecting out. The tumor mass was firmly impacted in the pelvis. There were no dense adhesions to adjacent structures. Total hysterectomy with bilateral salpingectomy was done. The buried uterus was exposed along with the mass only after clamping and cutting the uterine pedicles and vagina. The size of the mass was approximately 14 × 10 cms. The outer surface of the tumor showed numerous polypoidal masses, which on cut section was seen to arise from the cervix (Figure 3). Histopathology showed tumor arising from the cervix in the form of molded papillae lined by cuboidal epithelium and subepithelium showing scattered glands with surrounding stroma containing fibroblasts (Figure 4). The tumor does not appear to invade the cervical tissue. The diagnosis of cervical adenofibroma was made. Her postoperative recovery was uneventful, and she was discharged on the 14th day. She was healthy in her follow-up visits.

## 3. Discussion

Mixed epithelial-mesenchymal tumors contain both epithelial and mesenchymal elements as active participants in the neoplastic process. This tumor group includes adenofibroma and adenosarcoma. Women with uterine adenofibroma tend to be elderly and present with either abnormal uterine bleeding or postmenopausal bleeding [6]. There are a few reports of cervical adenofibroma in which the women were of older age groups and the size of the polypoidal mass was smaller [7, 8]. Our patient was a young woman, and the polypoidal mass was very large. She which presented with obstructive symptoms of acute retention of urine and absence of menstruation. The large size of the mass made it clinically difficult to locate the exact site of origin of the mass preoperatively. However, intraoperative finding of a cervical origin of the tumor mass might explain the obstructive symptoms in this case. Preoperative biopsy suggested the possibility of mixed epithelial and mesenchymal components of mullerian origin. The imaging techniques did not help to locate the exact origin of the tumor and to exclude malignancy in our patient. Although the unique sonographic finding of uterine adenofibroma was described, it was not diagnostic and inconclusive to exclude malignant component [7, 9].

It is important to distinguish adenofibroma from adenosarcoma. Adenofibroma contains a mixture of histologically bland epithelium and mesenchyme. It has broad papillary fronds covered by epithelium projecting from the surface of the neoplasm and extending into cystic spaces within it. Epithelium may be cuboidal as in this case. A mixture of various types of epithelia, including endocervical, tubal, and squamous often occur within the same neoplasm [5]. The mesenchymal component is usually benign homologous elements of fibroblast cells as in this case. The fibrotic stroma is more cellular and uniform than it is in polyps. Mitotic figures (MFs) are rare or invariably fewer than 4 MF/10 high power field [2]. Though this case has occasional MF, the absence of periglandular stromal hypercellularity, stromal cell atypia, and invasion distinguishes it from adenosarcoma.

Total hysterectomy is the preferred treatment for an adenofibroma because the neoplasm may recur if it is incompletely curetted or excised [10]. Total hysterectomy with bilateral salpingectomy, performed in our patient assured complete excision and adequate sampling to exclude malignancy. Though our patient, was a young unmarried lady, the option of conservative surgical resection was not considered for the following reasons. First, the biopsy specimen might not be a representative sample to exclude malignancy. Second, the large size of the tumor engulfing the uterus, firmly occupying the whole pelvis, made the conservative resection technically impossible. Third, the multiple recurrences as well as local invasion and involvement the adenocarcinoma have been reported with adenofibroma [10–12].

## 4. Conclusion

Adenofibroma should be considered in the differential diagnosis of uterine polyps even in young women. Thorough preoperative evaluation and complete surgical management is important to rule out malignancy and to prevent recurrences.
